# Elevated Cardiac Troponin in Non-Cardiac Conditions Unrelated to Acute Myocardial Infarction

**DOI:** 10.3390/ijms262311655

**Published:** 2025-12-01

**Authors:** Lidija Savic, Sanja Stankovic, Nebojsa Antonijevic, Dragan Matic, Ratko Lasica, Gordana Krljanac, Milika Asanin

**Affiliations:** 1 Faculty of Medicine, University of Belgrade, 11000 Belgrade, Serbia; drantoni@gmail.com (N.A.); dragan4m@gmail.com (D.M.); drlasica@gmail.com (R.L.); gkrljanac@gmail.com (G.K.); masanin2013@gmail.com (M.A.); 2 Cardiology Intensive Care Unit & Cardiology Clinic, Emergency Hospital, University Clinical Center of Serbia, 11000 Belgrade, Serbia; 3 Center for Medical Biochemistry, University Clinical Center of Serbia, 11000 Belgrade, Serbia; 4 Faculty of Medical Sciences, University of Kragujevac, 34000 Kragujevac, Serbia

**Keywords:** cardiac troponin, non-cardiac conditions, prognosis

## Abstract

Cardiac troponins (cTn) T and I are biochemical markers of myocardial injury. In this review article, we aim to summarize the mechanisms and significance of cardiac troponin (cTn) elevation unrelated to acute myocardial infarction (AMI) in the most frequently occurring non-cardiac conditions, where the accurate interpretation of elevated cTn levels is often challenging. Different mechanisms in non-cardiac conditions can cause non-ischemic myocardial injury. Understanding the pathophysiology of cTn release is an essential precondition for minimizing unnecessary, costly, and potentially risky (cardiac) interventions and for providing timely and appropriate medical care. Elevated cTn in critically ill patients and in patients with chronic disease/conditions is an independent predictor (risk factor) of cardiovascular and overall mortality. Treatment of underlying conditions is of primary importance, and close monitoring for the occurrence of cardiovascular complications during hospitalizations should be considered in these patients. Also, when the patient recovers from the underlying disease, clinical judgement should be employed to decide whether and to what extent further cardiological evaluation is indicated.

## 1. Introduction

Cardiac troponins T and I (cTnT and cTnI) are regulatory proteins located in cardiomyocytes that are released when cardiomyocyte injury occurs. Since cTnT and cTnI are expressed exclusively in myocardial tissue, their elevated levels in the serum are considered ideal markers for the detection of myocardial injury [1,2]. Myocardial injury can be acute or chronic [3,4]

Acute myocardial injury can be ischemic and non-ischemic and does not always lead to irreversible necrosis with scar formation [3,5]. Acute myocardial infarction (AMI) is one form of acute myocardial injury that is caused exclusively by ischemia. According to the Fourth Universal Definition of Myocardial Infarction, an elevated cardiac troponin (cTn) level is necessary but is not the only criterion for diagnosing AMI; at least two other criteria in addition to elevated cTn are required for establishing a diagnosis of AMI [4]. When there are sufficient criteria for diagnosing AMI, the next step is to categorize AMI as ST-segment elevation myocardial infarction (STEMI) or non-STEMI and then further distinguish it into five groups based on the mechanisms of ischemia [1,4,6,7]. Acute myocardial injury is frequently encountered in different clinical conditions and especially in critically ill patients. Although elevated cTn level in these patients may be caused by myocardial ischemia due to severe anemia or hypoxemia (the so-called type II AMI), it is most often caused by non-ischemic acute myocardial injury. Regardless of the cause, an elevated cTn level in acute myocardial injury is characterized by a rise and fall in the cTn level (the so-called dynamic change) [1,4].

Chronic myocardial injury can be found in some chronic conditions/diseases. It is characterized by a constant small increase (above the reference value) in the serum cTn level and the absence of dynamic change [1,4,8,9].

Emergency departments and intensive care units often test cTn values independently of patients’ symptoms or other clinical characteristics. According to data found in the literature, testing for cTn is ordered in 16.9% of patients presenting in emergency departments (ED) in the United States. However, chest pain or chest pain-equivalent symptoms account for approximately 5.3% of these patients [10]. Also, more than 50% of said patients with an elevated cTn level are without AMI and suffer from non-ischemic myocardial injury [3,6,11,12]. These data show that it is unnecessary to test cTn in many patients, especially those with low pre-test probability for the presence of coronary artery disease [13,14,15].

Understanding the mechanisms that lead to elevated serum cTn levels is important for the differential diagnosis of myocardial injury. If the increase in cTn is equated with AMI without considering other possible causes, a therapeutic problem may arise. Therapies approved for AMI are very often contraindicated and may actually harm patients with certain diagnoses, such as peptic ulcer, gastrointestinal bleeding, severe anemia, or intracranial hemorrhage. Also, by focusing solely on the diagnosis of coronary artery disease, the presence of another disease or condition that requires specific treatment (and wherein an increase in cTn, i.e., a non-ischemic myocardial injury, has occurred) may be overlooked. Although elevated cTn levels often present interpretative challenges in patients with non-cardiac and multifactorial conditions, an increased cTn value can almost always be correctly interpreted and explained through careful clinical evaluation of the patient [16,17,18,19,20,21,22,23].

This review aims to present the most important pathophysiological mechanisms leading to myocardial injury and the increase in serum cTn concentration and to discuss the significance of elevated cTn unrelated to myocardial infarction in different non-cardiac conditions.

## 2. Mechanism of cTn Release into the Serum

**Figure 1:**
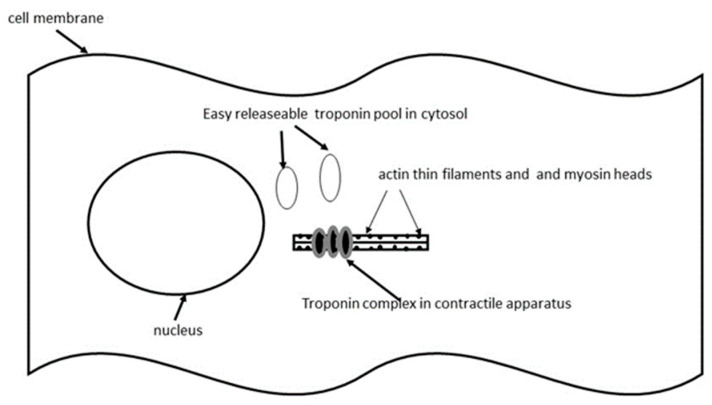
Troponins in cardiomyocytes.

Troponins are present in cardiomyocytes at two sites: in the contractile apparatus and in the cytosol of the cell (as shown in Figure 1), where only a small portion (about 5–8%) can be found. The cytosolic cTn pool, which is weakly bound to myofilaments, is called the *easily releasable pool*. From this easily releasable pool, cTn is readily released into the serum during normal cellular turnover (regeneration and renewal of myocardial cells), in conditions that increase cell membrane permeability (such as hypothyroidism), during myocardial strain or stress, and under strong sympathetic stimulation via beta-adrenergic receptors. The release of cTn from the easily releasable pool during normal cell turnover accounts for the small amount of circulating cTn (within the cut-off values) found in the serum of healthy individuals [2,24,25,26]. Mildly elevated cTn levels during minor, reversible myocardial injuries wherein the cell membrane remains intact (the so-called *leakage*) arise due to the activation of the calcium-sensitive protease calpain, which degrades cTn into smaller fragments. These degradation products, due to their reduced size, may exit from cells more easily and rapidly than the intact cTn molecule. Clinical conditions causing leakage of cTn from cardiomyocytes can sometimes lead to transient myocardial dysfunction [3,25,26,27,28]. Also, calpain activation may explain the modest and persistent elevation of cTn in serum which occurs in patients with systemic inflammatory processes [1,24].

If the process of myocardial tissue injury continues, in the next phase, degradation of the contractile apparatus (sarcomere) and cell necrosis occur, and a smaller or larger scar may form. Myofibrils are repeating units of Ca^2+^-activated ATP consuming sarcomere and constitute 50–60% of cardiomyocyte volume. If Ca^2+^ leaks in the cytoplasm, as a consequence of myocardial cell injury, the sarcomeres contract and consume all ATP, resulting in cardiomyocyte cell necrosis and release of cTn with other cellular contents [29].

Every acute myocardial injury is accompanied by a dynamic change in serum cTn levels (baseline value vs. subsequent value). An expert consensus group has recommended that serial changes greater than 20% be used to define significant change (e.g., cTn dynamics) [4,17]. An elevated serum cTn level after myocardial injury usually persists for 7–14 days, and sometimes it may persist for 7–21 days (so called troponin kinetics). This is particularly true when there is a larger zone of myocardial necrosis and/or when myocardial blood flow is not adequate [1,2]. There is no significant difference in cTn kinetics between patients with AMI and patients with acute non-ischemic myocardial injury [30]. Only in cases where there are scattered small zones of acute myocardial injury may elevated serum cTn levels be detected only a few days (and sometimes only 24 h) after the injury [2].

In conditions that lead to chronic myocardial injury, the dominant source of serum cTn is the easily releasable pool, while a significantly smaller amount is released through the degradation of the contractile apparatus, resulting in so-called micro-necroses without scar formation. In chronic myocardial injury, serum cTn levels are constant, i.e., without dynamic change, and can be continuously detected [4].

Finally, cardiac troponins or their fragments released into the bloodstream circulate for some time before being eliminated. The exact mechanism of cTn elimination is not completely understood, but it probably includes the reticuloendothelial system, cleavage of cTn molecules by proteolytic enzymes directly in the bloodstream, and elimination through glomerular filtration. There are also some data that show that cTnT and I are eliminated across blood–salivary barriers. The specific mechanism has not been determined, but this may be an explanation for the elevated cTn concentration in the oral fluid of patients with AMI [24]. The mechanisms that lead to cTn elevation in serum are presented in Table 1.

Basic facts about laboratory detection of cTn in the serum

High-sensitivity cTn assays (hs-Tn) for determining troponin levels are recommended by guidelines as they are more sensitive than standard assays; they allow detecting very low but diagnostically significant concentrations of cTn molecules in the blood serum [4,17,31,32,33,34]. High-sensitivity Tn assays differ from many other laboratory assays in that the assay-specific 99th percentile for healthy population, rather than a precisely defined upper cut-off value, serves as the diagnostic cut-off value for myocardial injury. Cardiac troponin levels in the serum with a concentration less than 99th percentile are considered normal myocardial metabolites [24]. The upper cut-off value, i.e., the 99th percentile for healthy individuals, indicates that, at the time it was defined, it excluded individuals with any cardiovascular or other diseases and conditions (including well-managed hypertension, smoking, use of various medications, etc.) and that it differs for each assay. There is some evidence that the 99th percentile value in the healthy population may vary depending on age and sex; however, adjusting the reference limits by age or sex is not currently recommended either in the biochemical or clinical approach [21,35].

Biochemical evidence indicates that injuries and diseases of the aorta and smooth muscle organs, such as those of the gastrointestinal system, urinary bladder, and skeletal muscles, can lead to the release of proteins into the serum that are detected as cTnT in laboratory tests. This explains elevated cTnT levels in cases of aortic dissection, bladder injury, ileus, and similar conditions [1,2,4,36]. On the other hand, increases in cTnI values have not been reported following the injury of non-cardiac tissues (the so-called cTnT/I mismatch) [4].

Finally, (rarely) falsely elevated cTn levels in the serum can be detected in the laboratory, and there are a few reasons for this. Biological interference in otherwise healthy individuals may occur if so-called macrotroponins or heterophilic autoantibodies are present in the serum. The presence of these molecules shifts the 99th percentile value for the healthy population toward higher levels. Routine testing for these autoantibodies is not recommended, except in cases of repeatedly mildly elevated hs-Tn values without clinical explanation [5,23,35]. The presence of rheumatoid factor in serum is detected in around 5% of healthy persons. Approximately 1% of healthy persons with positive rheumatoid factor in serum have elevated cTnI solely because of the rheumatoid factor, and rheumatoid-blocking agents can be used if this situation is suspected. Excess fibrin is another reason for falsely elevated cTnI levels. There are three possible biochemical approaches to correct this interference: heparinizing the tubes before analysis, removing excess fibrin by repeating centrifugation, and adding specific serum which contains protamine sulfate, thrombin and snake venom [5]. Interference with microparticles can also be avoided with repeated centrifugation. Finally, a malfunctioning analyzer can cause falsely elevated cTn levels [5]. The reasons for genuinely and falsely elevated cTn levels in the serum are shown in Table 2.

There are different assays for measuring hs-cTnI and one for measuring hs-cTnT levels. The analytical sensitivity (limit of detection) of hs-cTnI and hs-nT assays varies 10-fold, and values from one assay cannot be directly compared with those from another assay. Also, values may differ between assay generation, and changes can even occur when the same assay reagents are measured using different instruments. Although there are biochemical differences among them, in clinical practice, there are no significant differences, and detection of both hs-cTnI and hs-cTnT is used equally in everyday clinical practice, except in specific (and already known) clinical conditions such as skeletal muscle disorders/diseases (in which the hs-cTnI level should be determined to check if the heart is also involved) [4].

## 3. Pathophysiological Mechanisms and Clinical Conditions Other than AMI Causing Genuine hs-cTn Elevation

Acute and chronic clinical conditions that cause elevated cTn levels are presented in Table 2. They can be classified as cardiac conditions (other than acute myocardial ischemia) and other non-cardiac and multifactorial conditions. Non-ischemic myocardial injury in all of these diseases/conditions may result from a single pathophysiological mechanism but more often arises from a combination of several mechanisms. Although elevated cTn levels in the serum reflect acute and chronic myocardial cell injury, they do not indicate underlying pathophysiological mechanisms [4]. Non-cardiac conditions wherein cTn elevation can be found are presented in Table 3, and the mechanisms that lead to non-ischemic cTn elevation in serum are presented in Table 4.

### 3.1. Sepsis and Systemic Inflammatory Response Syndrome (SIRS)

Sepsis is a life-threatening condition characterized by a dysregulated immune response to infection, leading to multiple organ dysfunction, including cardiac involvement [37]. According to data from the literature, elevated serum hs-cTn levels are found in 36% to 85% of patients admitted to hospital with sepsis or systemic inflammatory response syndrome (SIRS). The varying prevalence of patients with elevated cTn values is most likely due to differences in the causative agents of sepsis, the varying degrees of immune response, and the use of different laboratory assays for determining serum cTn levels (high-sensitivity or standard assays) [37].

Information on chest pain is often unobtainable in patients with sepsis/SIRS (patients on mechanical ventilation, sedated patients, patients with delirium, etc.), and transient ECG changes, most commonly T-wave abnormalities, may be present. Although it is possible for a patient with sepsis/SIRS to develop AMI, in most patients with elevated cTn values after completion of sepsis treatment, angiography excludes the presence of significant coronary artery disease. In a study by Malomo et al., it was found that 27% of analyzed patients who survived sepsis and had left ventricular dysfunction had coronary artery disease on angiogram; 20% of analyzed patients had significant coronary artery stenosis (>70%); and 7% had moderate stenosis (50–70%). These findings indicate that acute myocardial injury in sepsis and/or SIRS is predominantly of non-ischemic origin [25,38].

**Figure 2:**
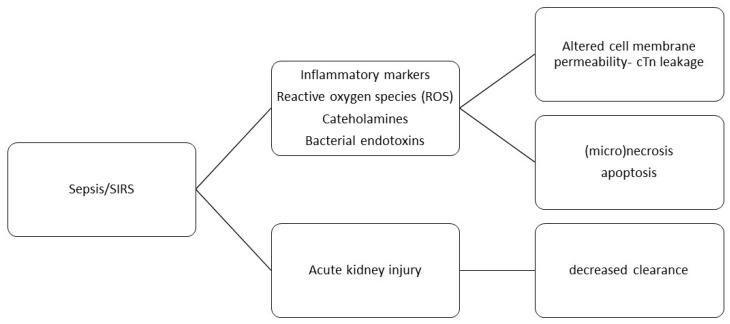
Mechanisms for cTn elevation in sepsis/SIRS.

Non-ischemic acute myocardial injury in sepsis/SIRS may occur for several reasons [1,11,25]. Elevated levels of circulating catecholamines and inflammatory cytokines, such as tumor necrosis factor-alpha (TNF-α), interleukin-1 (IL-1), and interleukin-6 (IL-6), can cause the formation of reactive oxygen species (ROS). ROS interact with phospholipids in cell membranes, altering membrane permeability and promoting the extracellular leakage of cytosolic proteins. Inflammatory cytokines TNF-alpha, IL-1, and IL-6 cause degradation of free cTn in cytosol (calpain activation) to low-molecular-weight fragments, which are then released into systemic circulation through the highly permeable cell membrane of cardiac myocytes [39]. The detection of these small cTn fragments may explain troponinemia in the absence of myocyte necrosis [1]. Furthermore, IL-6, TNF-α, and catecholamines induce a hypercatabolic state, stimulating muscle fiber proteolysis and necrosis most notably in skeletal muscle but also in cardiac myocytes, thereby contributing to overall catabolic stress. TNF-α can also cause cardiomyocyte apoptosis [39]. Bacterial endotoxins may exert direct cardiotoxic and/or cardiodepressive effects [40,41]. In addition, due to hemodynamic alterations, patients with sepsis/SIRS experience significant myocardial mechanical stretch, which can also increase cell membrane permeability without causing necrosis [4,7]. Finally, acute kidney injury (AKI) and exacerbation of chronic kidney disease, through their specific pathophysiological mechanisms, can lead to myocardial injury accompanied by cTn release [1]. The mechanisms of cTn release in patients with sepsis/SIRS are shown in Figure 2.

Patients exhibiting elevated cTn levels during sepsis or SIRS are frequently found to develop sepsis-induced cardiomyopathy. This condition is characterized by a spectrum of cardiac abnormalities, including left ventricular dilation, a reduced left ventricular ejection fraction (LVEF) with normal or increased filling pressures, and right ventricular dysfunction. In a study involving 58 consecutive patients admitted to the ICU with sepsis, all individuals with an LVEF < 45% demonstrated elevated cTn levels, with higher concentrations observed in those with a more severely reduced LVEF [37].

The therapeutic approach for patients with sepsis/SIRS, elevated cTn, and sepsis-induced cardiomyopathy primarily involves treating sepsis using evidence-based, guideline-directed therapy. Cardiac treatment is based on providing recommended treatments for any complications that may develop (e.g., heart failure, arrhythmias, etc.) [1]. Myocardial function usually recovers within 7–10 days if the underlying condition is resolved [4,39,40].

### 3.2. Elevated cTn Levels in Acute Neurological Conditions

Acute brain injury, including ischemic stroke and intracranial hemorrhage, may lead to elevated cTn levels as a result of acute myocardial injury [42,43,44,45,46]. Elevated cTn levels are observed in 30–60% of patients during the early phase of ischemic stroke and also in patients with subarachnoid hemorrhage [42,43]. Although official guidelines recommend measuring cTn levels in all patients with stroke, there are no clear guidelines on how to manage patients with elevated cTn or which further diagnostic workup, if any, should be performed [46].

Several factors can lead to elevated cTn levels in patients with acute brain injury: acute non-ischemic myocardial injury as part of stroke-heart syndrome, acute myocardial infarction (given that ischemic stroke and coronary artery disease share almost identical risk factors), and stress-induced cardiomyopathy (Takotsubo syndrome) [44]. However, coronary angiographic findings have shown that only a small percentage of patients with ischemic stroke and elevated cTn have significant coronary artery stenoses, indicating that the majority of cases are due to (non-ischemic) stroke-associated myocardial injury [43,46].

**Figure 3:**
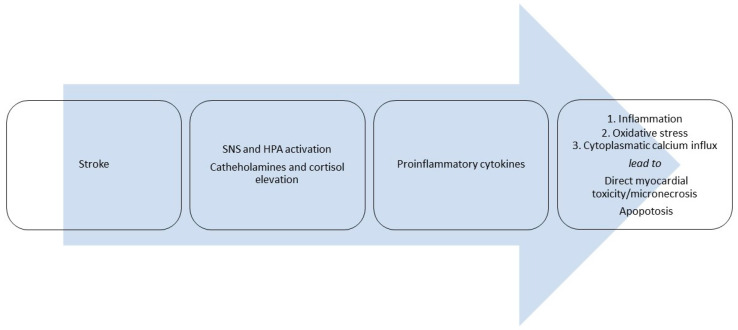
Mechanisms for non-ishemic myocardial injury in patients with stroke.

The most frequently observed pathophysiological mechanisms leading to stroke-associated myocardial injury are the following: a catecholamine surge caused by centrally mediated release of catecholamines in response to hypoperfusion of the posterior hypothalamus, and neurogenic cardiac damage in patients with stroke affecting the insular cortex, which plays a central role in the autonomic control of cardiac function [1,45]. Brain regions associated with specific cTnT elevations include the right posterior, superior, and medial insula, as well as the right inferior parietal lobe. Excessive catecholamine (and cortisol) levels lead to increased sarcoplasmic calcium influx, resulting in consecutive hypercontraction of sarcomeres, metabolic and oxidative stress, and electrical instability. There are also inflammatory responses associated with stroke. All these pathological processes can induce cardiomyocyte apoptosis, contraction band necrosis, and an interstitial inflammatory reaction in the myocardial tissue. Cardiac dysfunction, including transient wall motion abnormalities (other than stress-induced Takotsubo cardiomyopathy) and reduced ejection fraction (EF), is frequently observed in patients with acute stroke and other severe acute neurological conditions who have dynamic elevations of cTn [47]. The mechanisms of cTn release in patients with acute brain injury are shown in Figure 3.

Elevated cTn levels in patients with acute brain injury should always be interpreted with caution, and conclusions regarding the potential cause should be made only after a multidisciplinary assessment of the patient. Such an approach is absolutely necessary in this patient population because dual antiplatelet therapy carries a risk of secondary intracerebral hemorrhage in patients with ischemic stroke, and in young patients with intracranial hemorrhage who have a very low likelihood of coronary artery disease, the use of antiplatelet therapy is absolutely contraindicated [1,44]. Therapeutic options for this form of acute myocardial injury lack scientific evidence and are limited to symptomatic treatment [47].

### 3.3. Troponin Elevation in Acute and Chronic Kidney Disease (CKD)

Reduced kidney function, whether due to chronic kidney disease (CKD) or the development of acute kidney injury (AKI), is of particular importance when interpreting elevated cTn levels. It has been reported that over 50% of patients with severe CKD and over 75% of hemodialysis patients have elevated serum cTn of varying degrees. The mechanisms of cTn elevation are complex and, beyond reduced cTn clearance, likely include left ventricular hypertrophy, myocardial stretch, cardiomyocyte apoptosis, or direct toxic effects on cardiac and skeletal muscles associated with the uremic state [48,49,50]. Two studies found that cTn in serum in patients with CKD only existed in the free intact form (and not in the fragmented form) [51,52].

Numerous studies have shown a correlation between serum cTn levels and serum creatinine levels [33]. Furthermore, according to the majority of published analyses, in hemodialysis patients, cTn levels do not depend on the timing of dialysis, i.e., significant decreases or normalization of cTn are not observed after hemodialysis. Although some data indicate that cTn levels may slightly drop after hemodialysis, they do not reach the normal baseline values (defined by the 99th percentile of the healthy population) [2,48]. The methodology and type of dialysis may influence cTn levels by either resulting in more cardiac injury or actually clearing cTn molecules or fragmenting them, so they are no longer recognizable for laboratory assays [48].

In patients on hemodialysis, it has been observed that the elevation of cTnT levels is more frequent than the elevation of cTnI levels. This can be explained by a specific mechanism: cTnI, but not cTnT, adheres to the dialyzer membrane during hemodialysis. This phenomenon was first described with polysulfone dialyzer membranes, and since these membranes are not the only type used for hemodialysis, cTnI may also adhere to other types of synthetic dialysis membranes [3,5].

**Figure 4:**
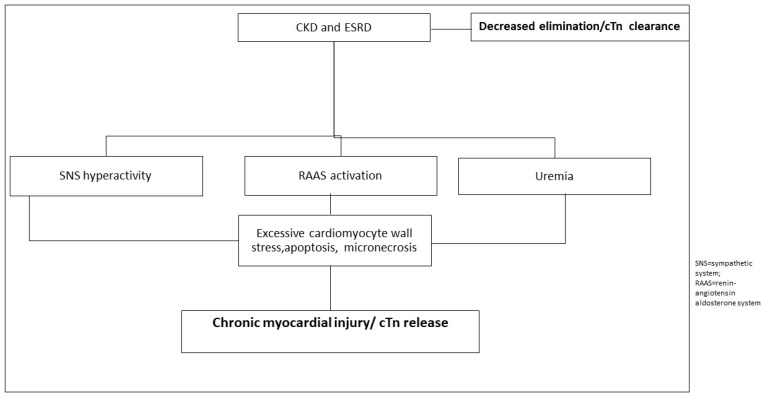
Presumed mechanisms for persistened cTn elevation in patients with CKD.

In addition to the kidneys, cTn can also be eliminated extrarenally via the reticuloendothelial system (RES) via scavenger receptors. Since not all patients with CKD have elevated cTn levels, and because the levels of elevated cTn vary among patients (with some showing higher and others lower values), it is believed that individual patients have differing extrarenal clearance via scavenger receptors. This phenomenon is likely due to genetic variations in scavenger receptors, and its significance remains a subject of further research [2]. Mechanisms for persistent cTn elevation in patients with CKD are presented in Figure 4.

### 3.4. Gastrointestinal Bleeding and Small Bowel Obstruction

Acute gastrointestinal bleeding (AGIB) can cause severe anemia, hypotension, and even hypovolemic shock. These complications can cause ischemic myocardial injury. Beside these, there are data indicating that severe AGIB can cause acute non-ischemic myocardial injury with transient loss of cardiac cell membrane integrity and with cTn elevation. Also, hypotension and hypovolemic shock in patients with severe AGIB cause acute kidney injury, which can also cause cTn elevation [53]. In patients with small bowel (ileus) obstruction, as already mentioned, elevated cTnT is detected. Elevated cTnI is detected if acute myocardial injury occurs, and it is caused in patients with ileus and sepsis/SIRS as a complication of the ileus [4,54].

### 3.5. Strenuous Exercise

Elevated cTnT and cTnI levels can be detected after strenuous and ultra-endurance exercise (e.g., long-distance running or triathlon) in almost all individuals (professional athletes and amateur runners or triathletes) [24,25,33,55,56]. Higher cTn levels have been registered in marathon runners who were men, were older in age, and had less athletic experience. Also, the degree of hs-cTn elevation was related to the intensity and duration of physical exertion [32].

It has been reported that elevated cTn levels upon intense exercise decline rapidly, 24 h after rest, and faster than cTn elevations caused by other forms of acute myocardial injury. The initially proposed mechanisms of this phenomenon include increased myocardial stress/strain with “leakage” of cTn from the easily releasable pool through intact cell membranes, excessive reactive oxygen species (ROS) production, acid–base imbalance, and passive transport of cTn from the intracellular to extracellular compartment, although the possibility of cardiomyocyte injury leading to apoptosis has also been suggested [25,33,56,57]. In addition to these mechanisms, so-called transient ischemic acute myocardial injury may also occur due to extremely high myocardial oxygen demand during strenuous exercise. The kinetics of cTn levels in serum after strenuous exercise differ from the kinetics seen in acute myocardial injury from other causes: after physical activity, elevated cTn levels persist for only 1–3 days [32].

### 3.6. Skeletal Muscle Disorders

In inflammatory skeletal muscle diseases, as well as in rhabdomyolysis, elevated cTnT levels can be detected in over 85% of patients, whereas cTnI levels are rarely registered. The initial explanation for these findings was that older cTnT laboratory assays exhibited cross-reactivity with certain troponin T epitopes released from skeletal muscle. However, even with the refinement of laboratory assays and the introduction of hs-cTnT, elevated cTn levels are still being detected in the absence of myocardial injury [57].

It is believed that the specific mechanism leading to the detection of hs-cTnT involves re-expression of the TNNT2 gene, which encodes cTnT in skeletal muscles that exhibit active disease on biopsy, as well as during tissue repair. This leads to the conclusion that an elevated cTnT level in patients with skeletal muscle disorders is genuine, i.e., it is not falsely elevated. However, the presence of a cTnT/I mismatch indicates that the source of the elevated cTnT is not myocardial tissue. This is why cTnI measurement is recommended for diagnosing myocardial injury in patients with skeletal muscle diseases and/or rhabdomyolysis [57].

### 3.7. Pulmonary Embolism

Around 30–60% of patients with pulmonary embolism (PE) have elevated cTn levels, and this depends on whether conventional or high-sensitive assays are used. Increased cTn levels are seen in high-risk and intermediate-high risk PE. The source of elevated serum cTn in patients with PE is the cardiac myocytes of the right ventricle. The main mechanism that causes acute myocardial injury in patients with PE is excessive neurohumoral activation that leads to massive infiltration of inflammatory cells in the right ventricular (RV) myocardium (this has been confirmed by autopsy in patients who died within 48 h of acute PE). Other reasons for cTn elevation include excessive right ventricular (RV) wall stress [58]. The kinetics of elevated cTn concentration in patients with PE differs from cTn kinetics in AMI; serum cTn reaches peak values after around 10 h, and the duration of elevated level is around 30–40 h [34].

### 3.8. Systemic Autoimmune and Infiltrative Diseases

Many systemic autoimmune diseases affect the myocardium and cause autoimmune myocarditis, which is the most common cause of elevated cTn level in these patients. The prevalence of patients with myocardial involvement as part of systemic autoimmune diseases is about 30% in patients with polymyositis, about 5% in patients with systemic systemic lupus erythematosus, and about 25% in patients with sarcoidosis. In all these diseases, the most common mechanism for cTn release is cell necrosis caused by myocardial inflammation, and the magnitude of cTn elevation is crucial in diagnosing the severity of myocarditis. Some diseases can also have other specific mechanisms for myocardial injury; in patients with antiphospholipid syndrome, myocardial damage can be caused not only by inflammation but also by microthrombosis. Structural damage of the vascular bed leading to repeated focal ischemic injury and irreversible myocardial fibrosis is common in patients with systemic sclerosis [59]. In patients with infiltrative diseases such as amyloidosis, direct cardiomyocyte compression and subsequent damage is the probable cause of cTn elevation [3].

### 3.9. Acute Allograft Rejection

Acute cellular rejection (ACR) after heart transplantation occurs in up to 30% of patients in the first year. Since direct myocardial cell damage and necrosis are a hallmark of ACR, elevated cTn levels are seen during a significant episode of ACR. On the other hand, there are patients with elevated cTn levels who do not have ACR on endomyocardial biopsy. The pathophysiological mechanisms that are responsible for cTn serum elevation in patients without EMB-proven ACR are not fully understood [60]. In patients after heart transplantation, the cardiac Tn level in the reference range may have sufficient sensitivity to exclude ACR. On the other hand, elevated cTn levels do not have sufficient specificity to replace endomyocardial biopsy in patients with suspected ACR [60].

### 3.10. Chemotherapeutic Agents

Many medications, especially chemotherapeutics and antineoplastic agents, have an adverse effect on cardiac myocytes and can cause different cardiovascular complications. A comprehensive review of all cardiotoxic medications is beyond the scope of this paper, and we will mention only the most commonly implicated drugs; these are antrhacyclines and trastuzumab, which is monoclonal antibody directed against human epidermal growth factor receptor 2 (HER-2). Antrhacyclines injure cardiac myocytes primarily by inhibiting topoisomerase-2 beta which increases breakage of double-strand DNA in addition to increasing free radical production via iron deposition in mitochondria. Another mechanism for myocardial cell injury is an increase in the intracellular calcium level, which can cause direct cell injury. All these mechanisms lead to myocardial cell apoptosis, necrosis, and cardiomyopathy. There is a direct correlation between cumulative dose of anthracyclines, magnitude of cTn elevation, and myocardial damage. Trastuzumab can also be cardiotoxic, especially in combination with anthracyclines. Anthracycline-induced oxidative damage leads to upregulation of HER-2 receptors as a result of cellular repair. Inhibition of HER-2 receptor in combination with anthracycline effects can cause significant myocardial cell necrosis and cardiomyopathy. Irrespective of treatment with anthracycline, trastuzumab itself can cause myocardial cell damage and cardiomyopathy, but it is usually reversible [1]. Cardiac Tn level measurement prior to and during administration of cardiotoxic medications (together with cardiac imaging, i.e., transthoracic echocardiography) is necessary for early detection of patients who are at high risk of developing drug-induced cardiomyopathy [1].

## 4. The Clinical Significance of cTn Elevation in Patients with Non-Cardiac Diseases

Critically ill patients and patients with sepsis/SIRS who have elevated cTn levels usually present with multiple comorbidities and a higher Charlson comorbidity index compared with patients with the same conditions but without elevated cTn. Their hemodynamic status is worse in terms of more frequent hypotension, tachycardia, and hypoxemia. On ECG, atrial fibrillation or non-specifically prolonged QRS complexes are more commonly observed [12,16].

Elevated cTn in critically ill patients and those with sepsis/SIRS reflects the severity of the underlying disease and serves as an independent predictor of both in-hospital and long-term mortality [6,11,16,20,29,33,61,62,63,64,65,66,67,68,69,70]. In a study by Lim et al. [66], cTn was elevated in 43% of non-cardiac ICU patients, and these patients had a 250% higher mortality compared with patients without elevated cTn levels [67]. In patients with sepsis/SIRS, thirty-day mortality was four times higher in those with elevated cTn levels, as compared to patients whose cTn levels were within the reference range [63]. This finding is expected, as elevated cTn in patients with sepsis indicates the development of sepsis-induced cardiomyopathy. Since elevated cTn is a biomarker of poor prognosis in critically ill patients, a question has been raised regarding the practical application of this finding and the use of cTn in improving risk stratification for these patients. Studies have shown that higher cTn levels are associated with increased Sequential Organ Failure Assessment (SOFA) and Acute Physiology and Chronic Health Evaluation (APACHE) II scores in critically ill patients admitted to the ICU [16,26]. These findings initially suggested that incorporating elevated cTn levels might improve risk prediction models for critically ill patients [33,66,67,68]. However, adding cTnI values to the APACHE II score did not enhance its predictive performance [26]. Given the cost of testing and the lack of additional benefit compared to existing prediction models, routine measurement of cTn in all ICU patients is not recommended. If there is any suspicion of developing cardiac complications in critically ill patients—such as unexplained hemodynamic instability or the occurrence of arrhythmias—measuring serum cTn levels is justified, since these patients may benefit from closer hemodynamic monitoring, more aggressive treatment of the underlying condition, and the introduction of cardiac medications such as beta-blockers or ACE inhibitors, when clinically feasible [26].

In patients with ischemic stroke, elevated cTn levels are associated with poorer functional outcomes, more than a twofold increase in mortality, and a higher risk of major cardiovascular events during both short- and long-term follow-up. Moreover, elevated hs-cTn levels are also linked to the degree of severity of cerebral small vessel disease and impaired cognitive function. Elevated cTn levels in patients with acute ischemic stroke may be associated with left ventricular systolic dysfunction and are also an independent predictor of mortality [47]. Similar findings are reported among patients with subarachnoidal hemorrhage. One retrospective study of 617 consecutive patients showed increased mortality in patients with elevated cTnI levels (as compared with patients with no cTnI elevation) [69]. All patients with elevated cTn and ischemic stroke should undergo 24 h Holter ECG monitoring and echocardiography. Given that patients with ischemic stroke are generally at high risk of cardiovascular disease, they require continued follow-up by both a cardiologist and a neurologist. A similar approach is recommended for patients with intracranial hemorrhage and elevated hs-cTn levels [43].

In patients with CKD, persistently elevated hs-cTn is a predictor of many adverse events, including incident heart failure, cardiovascular mortality, and all-cause mortality [33]. Previous analyses have demonstrated the negative prognostic impact of elevated cTn levels in patients with CKD and a significant correlation with left ventricular systolic dysfunction [44]. The large National Institutes of Health (NIH)-sponsored multicenter Chronic Renal Insufficiency Cohort (CRIC) study, which included 3243 well-characterized subjects, provided the most definitive data regarding baseline hs-cTnT levels, their cross-sectional associations with cardiovascular risk factors, echocardiographic evidence of cardiac abnormalities, and longitudinal outcomes [70,71,72,73]. In this cohort, hs-cTnT was detectable in 84% of CKD patients; higher levels were strongly and independently associated with left ventricular hypertrophy (LVH) and, to a lesser extent, with left ventricular systolic dysfunction, as assessed by echocardiography [70]. Longitudinal outcomes from the CRIC study showed increased incident heart failure (adjusted HR 4.77, 95% CI 2.49–9.14) when comparing patients with normal hs-cTnT levels to those in the highest quartile of hs-cTnT after a median follow-up of six years [68,69]. These findings confirmed the results of the earlier large PREVEND observational study involving 1505 patients, mostly with mild CKD, which reported similar findings, although it lacked detailed structural cardiac data apart from LVH defined by ECG criteria. In the PREVEND study, elevated hs-cTnT remained a significant prognostic marker of cardiovascular events, after adjustment for demographics, kidney function, urinary albumin excretion, cardiovascular risk factors, and ECG abnormalities [72,73]. Additionally, in patients undergoing kidney transplantation, elevated pre-transplant cTn levels have been shown to correlate with an increased risk of post-transplant mortality and adverse cardiovascular events, independent of other risk factors [74]. Overall, cardiac troponins may be valuable in the management of CKD patients by aiding in more accurate cardiovascular risk stratification [32].

In patients with AGIB, cTn elevation is a risk factor for mortality, longer hospital stay, and need for urgent endoscopic evaluation as compared with patients with no cTn elevation [53]. In patients with small bowel obstruction, elevated cTnI is associated with worse outcomes, highlighting the need for prompt surgical assessment [54].

The diagnostic value of elevated cTn levels following physical exertion remains controversial, and discussions on this topic are still ongoing. Elevated cTn levels after intense exercise more likely reflect the heart’s adaptation to significant and prolonged physical exertion rather than permanent cardiac damage [55,56]. Studies suggest that the rise in cTn is caused by transient myocardial injury, as cardiac magnetic resonance imaging (MRI) with gadolinium-based contrast agents has shown no evidence of cardiomyocyte necrosis and fibrosis with scar formation [33,55]. However, as a result of repeated episodes of myocardial injury and insufficient recovery (in professional athletes), progressive myocardial remodeling may occur [55]. Detailed cardiological evaluations performed after ultra-endurance physical activity have revealed structural cardiac changes in many athletes and, less frequently, previously undiagnosed coronary artery disease in older professional athletes (above the age of 35) who had previously been considered healthy. As such, any elevation of cTn following physical activity should be thoroughly investigated. The patient should be briefly hospitalized to allow for the possible detection of subclinical heart disease that may have become unmasked as a result of intense physical exertion [25,33,55].

Elevated cTn in patients with PE is associated with right ventricular dysfunction, and in hypotensive patients, short-term mortality is increased by 2–5 times as compared with normotensive patients [34]. In normotensive patients with acute PE, increased cTn levels alone have relatively low specificity and positive predictive value for early mortality. However, when combined with clinical and imaging findings in normotensive patients, elevated cTn levels can improve the identification of patients who have an elevated PE-risk and in whom thrombolytic therapy should be considered [58].

In patients with systemic autoimmune disorders, elevated troponin values indicate not only myocardial involvement but also disease activity. Elevated cTn levels have occasionally been found even in the absence of obvious cardiac symptoms. In patients with rheumatoid arthritis, elevated cTn levels correlate with (elevated) TNF levels. In asymptomatic patients with systemic lupus erythematosus, elevated cTn levels have been associated with cardiac magnetic resonance imaging findings of myocardial edema. In a study by Baba et al. [75] which included patients with sarcoidosis, elevated cTn levels were associated with long-term adverse outcomes (which included the composite end point of heart failure death, sudden cardiac death, and hospitalization for heart failure), even if cardiac involvement was not detected using different imaging modalities (at the time when patients were included in analysis). This finding indicates that patients might already have been suffering from latent cardiac involvement, and cTn can be a promising biomarker for cardiac screening and a predictor of cardiac adverse events in patients with sarcoidosis [75,76]. An elevated value of cTn in patients with autoimmune diseases requires urgent referral to a cardiologist for additional diagnostics, and this is absolutely necessary for these patients. Therapy primarily involves treatment of the underlying autoimmune disease with supportive cardiac therapy [77].

Finally, studies have found that elevated baseline cTnT levels and changes in cTnT levels measured with a highly sensitive assay are also significantly associated with incident HF and cardiovascular death in the general population and in older people without previous cardiovascular disease [31,32,33,78].

## 5. Conclusions

Myocardial injury accompanied by significant hs-cTn elevation is often detected in patients with non-cardiac and multifactorial conditions. Numerous mechanisms other than ischemia can lead to acute myocardial injury in such cases. Therefore, an elevated cTn level should not be regarded as synonymous with acute myocardial infarction. A thorough understanding of the pathophysiology of cTn release is an essential prerequisite for minimizing unnecessary, costly, and potentially risky (cardiac) interventions while ensuring timely and appropriate medical care directed toward the primary (underlying) condition or disease. Elevated cTn in critically ill patients and in patients with chronic diseases/conditions is a risk factor for cardiovascular and overall mortality. Treatment of the underlying condition or disease remains the primary priority, and close monitoring for the development of cardiovascular complications during hospitalization should be considered in these patients. Furthermore, once the patient has recovered from the underlying illness, clinical judgment should be employed to determine whether, and to what extent, additional cardiologic evaluation is indicated.

## Figures and Tables

**Table 1:** Mechanisms that lead to cTn elevation in serum.

Increased membrane permeability
Myocardial cell necrosis
Myocardial cell apoptosis
Decreased cTn clearance (elimination)

**Table 2:** Reasons for laboratory detection of elevated hs-cTn in the serum (above the 99th percentile).

Genuinely Elevated
cTn leakage from the easily releasable pool
Release from the myofibril-bound troponin pool (apoptosis, necrosis)
**False positive/elevated**
Presence of heterophilic antibodies in the serum
Rheumatoid factor in the serum
Microparticles
Hemolysis
Elevated alkaline phosphatase
Fibrin clot in the serum
Malfunction of the analyzer

**Table 3:** Non-cardiac conditions that can present with genuinely elevated cTn levels (unrelated to myocardial infarction).

Critically Ill Patients
Sepsis/septic shock/systemic inflammatory response syndrome (SIRS)
Acute pancreatitis
Burns (affecting >30% of body surface area)
Acute brain injury—ischemic stroke, subarachnoid hemorrhage, intracranial hemorrhage
Chronic kidney disease
Acute kidney injury
Acute gastrointestinal tract (GIT) conditions (e.g., gastrointestinal bleeding, ileus)
Pulmonary embolism and pulmonary hypertension
Systemic autoimmune disease
Infiltrative disease (e.g., amyloidosis, sarcoidosis)
Chemotherapeutic agents
Hyper- or hypothyroidism
Strenuous exercise
Neuromuscular disease (only cTnT)
Myositis (only cTnT)
Rhabdomyolysis (only cTnT)

**Table 4:** Mechanisms of non-ischemic myocardial injury that lead to cTn elevation in conditions presented in Table 3.

Direct Cell Damage
Catecholamine excess (via beta1-adrenergic receptors)
Increased ventricular wall tension and strain
Myocyte trauma

## Data Availability

No new data were created or analyzed in this study. Data sharing is not applicable to this article.

## References

[1] Sternberg M., Pasini E., Chen-Scarabelli C., Corsetti G., Patel H., Linardi D., Onorati F., Faggian G., Scarabelli T., Saravolatz L. (2019). Elevated Cardiac Troponin in Clinical Scenarios Beyond Obstructive Coronary Artery Disease. Med. Sci. Monit..

[2] Hammarsten O., Mair J., Möckel M., Lindahl B., Jaffe A.S. (2018). Possible mechanisms behind cardiac troponin elevations. Biomarkers.

[3] Park K.C., Gaze D.C., Collinson P.O., Marber M.S. (2017). Cardiac troponins: From myocardial infarction to chronic disease. Cardiovasc. Res..

[4] Thygesen K., Alpert J.S., Jaffe A.S., Chaitman B.R., Bax J.J., Morrow D.A., White H.D., ESC Scientific Document Group (2019). Fourth universal definition of myocardial infarction (2018). Eur. Heart J..

[5] Roongsritong C., Warraich I., Bradley C. (2004). Common causes of troponin elevations in the absence of acute myocardial infarction: Incidence and clinical significance. Chest.

[6] Meigher S., Thode H.C., Peacock W.F., Bock J.L., Gruberg L., Singer A.J. (2016). Causes of Elevated Cardiac Troponins in the Emergency Department and Their Associated Mortality. Acad. Emerg. Med..

[7] Chapman A.R., Taggart C., Boeddinghaus J., Mills N.L., Fox K.A.A. (2025). Type 2 myocardial infarction: Challenges in diagnosis and treatment. Eur. Heart J..

[8] Wang X.Y., Zhang F., Zhang C., Zheng L.R., Yang J. (2020). The Biomarkers for Acute Myocardial Infarction and Heart Failure. Biomed. Res. Int..

[9] Wildi K., Twerenbold R., Mueller C. (2015). How acute changes in cardiac troponin concentrations help to handle the challenges posed by troponin elevations in non-ACS-patients. Clin. Biochem..

[10] Makam A.N., Nguyen O.K. (2015). Use of cardiac biomarker testing in the emergency department. JAMA Intern. Med..

[11] Oh A.R., Lee S.H., Park J., Choi D.C., Yang K. (2023). Association between cardiac troponin testing at scheduled admission and mortality in patients with comorbidities. Ann. Transl. Med..

[12] Bardají A., Cediel G., Carrasquer A., de Castro R., Sánchez R., Boqué C. (2015). Troponin elevation in patients without acute coronary syndrome. Rev. Esp. Cardiol..

[13] Fihn S.D., Gardin J.M., Abrams J., Berra K., Blankenship J.C., Dallas A.P., Douglas P.S., Foody J.M., Gerber T.C., Hinderliter A.L. (2012). 2012 ACCF/AHA/ACP/AATS/PCNA/SCAI/STS guideline for the diagnosis and management of patients with stable ischemic heart disease: A report of the American College of Cardiology Foundation/American Heart Association task force on practice guidelines, and the American College of Physicians, American Association for Thoracic Surgery, Preventive Cardiovascular Nurses Association, Society for Cardiovascular Angiography and Interventions, and Society of Thoracic Surgeons. Circulation.

[14] Newby L.K., Jesse R.L., Babb J.D., Christenson R.H., De Fer T.M., Diamond G.A., Fesmire F.M., Geraci S.A., Geraci S.A., Geraci S.A. (2012). ACCF 2012 expert consensus document on practical clinical considerations in the interpretation of troponin elevations: A report of the American College of Cardiology Foundation task force on Clinical Expert Consensus Documents. J. Am. Coll. Cardiol..

[15] Penumetsa S.C., Mallidi J., Friderici J.L., Hiser W., Rothberg M.B. (2012). Outcomes of patients admitted for observation of chest pain. Arch. Intern. Med..

[16] Akbas T. (2024). Elevated Cardiac Troponin Levels as a Predictor of Increased Mortality Risk in Non-Cardiac Critically Ill Patients Admitted to a Medical Intensive Care Unit. J. Clin. Med..

[17] Mair J., Lindahl B., Müller C., Giannitsis E., Huber K., Möckel M., Plebani M., Thygesen K., Jaffe A.S. (2018). What to do when you question cardiac troponin values. Eur. Heart J. Acute Cardiovasc. Care.

[18] Wildi K., Gimenez M.R., Twerenbold R., Reichlin T., Jaeger C., Heinzelmann A., Arnold C., Nelles B., Druey S., Haaf P. (2015). Misdiagnosis of Myocardial Infarction Related to Limitations of the Current Regulatory Approach to Define Clinical Decision Values for Cardiac Troponin. Circulation.

[19] Wildi K., Gimenez M.R., Boeddinghaus J., Nestelberger T., Lopez-Ayala P., Koechlin L., Gerstenberger M., Carter N., Bima P., Glaeser J. (2025). Possible Misdiagnosis of Myocardial Infarction Using Regulatory-Approved and Close-to-Bioequivalent Upper Limits of Normal for Cardiac Troponin. J. Am. Heart Assoc..

[20] Nseir M., Mokhtari A., Stanisic M., Ekström U., Labaf A. (2024). Validation and correlation of high-sensitive troponin I and troponin T in the emergency department. BMC Cardiovasc. Disord..

[21] Sayed Masri S.N.N., Basri F., Yunus S.N., Cheah S.K. (2025). Cardiac Troponin as a Prognostic Indicator for Major Adverse Cardiac Events in Non-Cardiac Surgery: A Narrative Review. Diagnostics.

[22] Netala V.R., Hou T., Wang Y., Zhang Z., Teertam S.K. (2025). Cardiovascular Biomarkers: Tools for Precision Diagnosis and Prognosis. Int. J. Mol. Sci..

[23] Salvatici M., Sommese C., Corsi Romanelli M.M., Drago L. (2025). Review of Literature and Recommended Procedures for Management of Unusual Cases of False Positive Troponin Tests. Int. J. Mol. Sci..

[24] Chaulin A.M. (2021). Cardiac Troponins Metabolism: From Biochemical Mechanisms to Clinical Practice (Literature Review). Int. J. Mol. Sci..

[25] Zaki H.A., Shaban A.E., Shaban A.E., Shaban E.E. (2022). Interpretation of Cardiac and Non-Cardiac Causes of Elevated Troponin T Levels in Non-Acute Coronary Syndrome Patients in the Emergency Department. Cureus.

[26] Cole E.M., Docherty A.B. (2020). Troponin in critical care patients and outcomes. Br. J. Hosp. Med..

[27] Sandoval Y., Jaffe A.S. (2019). Type 2 Myocardial Infarction: JACC Review Topic of the Week. J. Am. Coll. Cardiol..

[28] Morrow D.A., Cannon C.P., Jesse R.L., Newby L.K., Ravkilde J., Storrow A.B., Wu A.H.B., Christenson R.H., Apple F.S., Francis G. (2007). National Academy of Clinical Biochemistry. National Academy of Clinical Biochemistry Laboratory Medicine Practice Guidelines: Clinical characteristics and utilization of biochemical markers in acute coronary syndromes. Circulation.

[29] Piper H.M., Meuter K., Schäfer C. (2003). Cellular mechanisms of ischemia-reperfusion injury. Ann. Thorac. Surg..

[30] Wereski R., Kimenai D.M., Taggart C., Doudesis D., Lee K.K., Lowry M.T.H., Bularga A., Lowe D.J., Fujisawa T., Apple F.S. (2021). Cardiac Troponin Thresholds and Kinetics to Differentiate Myocardial Injury and Myocardial Infarction. Circulation.

[31] van der Linden N., Klinkenberg L.J.J., Bekers O., Loon L.J.C.V., Dieijen-Visser M.P.V., Zeegers M.P., Meex S.J.R. (2016). Prognostic value of basal high-sensitive cardiac troponin levels on mortality in the general population: A meta-analysis. Medicine.

[32] McEvoy J.W., Daya N., Tang O., Fang M., Ndumele C.E., Coresh J., Christenson R.H., Selvin E. (2023). High-sensitivity troponins and mortality in the general population. Eur. Heart J..

[33] Aimo A., Georgiopoulos G., Panichella G., Vergaro G., Passino C., Emdin M., Clerico A. (2022). High-sensitivity troponins for outcome prediction in the general population: A systematic review and meta-analysis. Eur. J. Intern. Med..

[34] Chauin A. (2021). The Main Causes and Mechanisms of Increase in Cardiac Troponin Concentrations Other Than Acute Myocardial Infarction (Part 1): Physical Exertion, Inflammatory Heart Disease, Pulmonary Embolism, Renal Failure, Sepsis. Vasc. Health Risk Manag..

[35] Aakre K.M., Saenger A.K., Body R., Collinson P., Hammarsten O., Jaffe A.S., Kavsak P., Omland T., Ordonez-Lianos J., Apple F.S. (2022). Analytical Considerations in Deriving 99th Percentile Upper Reference Limits for High-Sensitivity Cardiac Troponin Assays: Educational Recommendations from the IFCC Committee on Clinical Application of Cardiac Bio-Markers. Clin. Chem..

[36] Kajioka S., Takahashi-Yanaga F., Shahab N., Onimaru M., Matsuda M., Takahashi R., Asano H., Morita H., Morimoto S., Yonemitsu Y. (2012). Endogenous cardiac troponin T modulates Ca^2+^-mediated smooth muscle contraction. Sci. Rep..

[37] Shvilkina T., Shapiro N. (2023). Sepsis-Induced myocardial dysfunction: Heterogeneity of functional effects and clinical significance. Front. Cardiovasc. Med..

[38] Malomo S., Oswald T., Alway T., Hadjivassilev S., Coombs S., Ellery S., Lee J., Phillips C., Philips B., James R. (2025). Characterization of Coronary Artery Disease in Sepsis Survivors. Biomedicines.

[39] Pasini E., Corsetti G., Aquilani R., Romano C., Picca A., Calvani R., Dioguardi F.S. (2018). Protein-Amino Acid Metabolism Disarrangements: The Hidden Enemy of Chronic Age-Related Conditions. Nutrients.

[40] Sato R., Nasu M. (2015). A review of sepsis-induced cardiomyopathy. J. Intensive Care.

[41] Antonucci E., Fiaccadori E., Donadello K., Taccone F.S., Franchi F., Scolletta S. (2014). Myocardial depression in sepsis: From pathogenesis to clinical manifestations and treatment. J. Crit. Care.

[42] Zhang L., Wang Z., Qi S. (2015). Cardiac Troponin Elevation and Outcome after Subarachnoid Hemorrhage: A Systematic Review and Meta-analysis. J. Stroke Cerebrovasc. Dis..

[43] Kruska M., Fastner C., Scheitz J.F., Kolb A., Rutsch M., Papavassiliu T., Borggrefe M., Alonso A., Akin I., Szabo K. (2021). Troponinerhöhung beim akuten ischämischen Schlaganfall—Unspezifisch oder Ausdruck eines akuten Myokardinfarkts?: Diagnostik und klinische Implikationen [Troponin elevation in acute ischemic stroke-unspecific or acute myocardial infarction?: Diagnostics and clinical implications]. Herz.

[44] Scheitz J.F., Stengl H., Nolte C.H., Landmesser U., Endres M. (2021). Neurological update: Use of cardiac troponin in patients with stroke. J. Neurol..

[45] Krishnamoorthy V., Mackensen G.B., Gibbons E.F., Vavilala M.S. (2016). Cardiac Dysfunction After Neurologic Injury: What Do We Know and Where Are We Going?. Chest.

[46] Stengl H., Ganeshan R., Hellwig S., Blaszczyk E., Fiebach J.B., Nolte C.H., Bauer A., Schulz-Menger J., Endres M., Scheitz J.F. (2021). Cardiomyocyte Injury Following Acute Ischemic Stroke: Protocol for a Prospective Observational Cohort Study. JMIR Res. Protoc..

[47] Xu C., Zheng A., He T., Cao Z. (2020). Brain-Heart Axis and Biomarkers of Cardiac Damage and Dysfunction after Stroke: A Systematic Review and Meta-Analysis. Int. J. Mol. Sci..

[48] Parikh R.H., Seliger S.L., de Filippi C.R. (2015). Use and interpretation of high sensitivity cardiac troponins in patients with chronic kidney disease with and without acute myocardial infarction. Clin. Biochem..

[49] Long B., Belcher C.N., Koyfman A., Bronner J.M. (2020). Interpreting troponin in renal disease: A narrative review for emergency clinicians. Am. J. Emerg. Med..

[50] Cyon L., Kadesjö E., Edgren G., Roos A. (2024). Acute Kidney Injury and High-Sensitivity Cardiac Troponin T Levels in the Emergency Department. JAMA Netw. Open.

[51] Bates K.J., Hall E.M., Fahie-Wilson M.N., Kindler H., Bailey C., Lythall D., Lamb E.J. (2010). Circulating immunoreactive cardiac troponin forms determined by gel filtration chromatography after acute myocardial infarction. Clin. Chem..

[52] Fahie-Wilson M.N., Carmichael D.J., Delaney M.P., Stevens P.E., Hall E.M., Lamb E.J. (2006). Cardiac troponin T circulates in the free, intact form in patients with kidney failure. Clin. Chem..

[53] Iqbal U., Siddique O., Jameel A., Anwar H., Chaudhary A. (2017). Prognostic Significance of Elevated Cardiac Troponin in Acute Gastrointestinal Bleeding. Gastroenterol. Res..

[54] Kumar M., Kayee C., Bhuvana J.A., Li C.H., Humes D. (2025). Troponin Elevation in Small Bowel Obstruction: Myth or Reality?. BJS.

[55] Janik M., Blachut D., Czogalik Ł., Tomasik A.R., Wojciechowska C., Kukulski T. (2025). Adaptive Changes in Endurance Athletes: A Review of Molecular, Echocardiographic and Electrocardiographic Findings. Int. J. Mol. Sci..

[56] Wuestenfeld J.C., Kastner T., Hesse J., Fesseler L., Frohberg F., Rossbach C., Wolfarth B. (2024). Differences in Troponin I and Troponin T Release in High-Performance Athletes Outside of Competition. Int. J. Mol. Sci..

[57] du Fay de Lavallaz J., Prepoudis A., Wendebourg M.J., Kesenheimer E., Kyburz D., Daikeler T., Haaf P., Wanschitz J., Löscher W.N., Schreiner B. (2022). BASEL XII Investigators. Skeletal Muscle Disorders: A Noncardiac Source of Cardiac Troponin T. Circulation.

[58] Konstantinides S.V., Meyer G., Becattini C., Bueno H., Geersing G.J., Harjola V.P., Huisman M.V., Humbert M., Jennings C.S., Jiménez D. (2020). 2019 ESC Guidelines for the diagnosis and management of acute pulmonary embolism developed in collaboration with the European Respiratory Society (ERS). Eur. Heart J..

[59] Schulz-Menger J., Collini V., Gröschel J., Adler Y., Brucato A., Christian V., Ferreira V.M., Gandjbakhch E., Heidecker B., Kerneis M. (2025). 2025 ESC Guidelines for the management of myocarditis and pericarditis: Developed by the task force for the management of myocarditis and pericarditis of the European Society of Cardiology (ESC)Endorsed by the Association for European Paediatric and Congenital Cardiology (AEPC) and the European Association for Cardio-Thoracic Surgery (EACTS). Eur. Heart J..

[60] Fitzsimons S., Evans J., Parameshwar J., Pettit S.J. (2018). Utility of troponin assays for exclusion of acute cellular rejection after heart transplantation: A systematic review. J. Heart Lung Transplant..

[61] Árnadóttir Á., Vestergaard K.R., Pallisgaard J., Sölétormos G., Steffensen R., Goetze J.P., Iversen K. (2018). High-sensitivity cardiac troponin T is superior to troponin I in the prediction of mortality in patients without acute coronary syndrome. Int. J. Cardiol..

[62] Kaldal A., Tonstad S., Jortveit J. (2023). Association of Troponin T measurements with long-term outcomes in patients with coronary artery disease participating in a secondary prevention trial. BMC Cardiovasc. Disord..

[63] Kim J.S., Kim M., Kim Y.J., Ryoo S.M., Sohn C.H., Ahn S., Kim W.Y. (2019). Troponin Testing for Assessing Sepsis-Induced Myocardial Dysfunction in Patients with Septic Shock. J. Clin. Med..

[64] Vallabhajosyula S., Sakhuja A., Geske J.B., Kumar M., Poterucha J.T., Kashyap R., Kashani K., Jaffe A.S., Jentzer J.C. (2017). Role of Admission Troponin-T and Serial Troponin-T Testing in Predicting Outcomes in Severe Sepsis and Septic Shock. J. Am. Heart Assoc..

[65] Bessière F., Khenifer S., Dubourg J., Durieu I., Lega J.C. (2013). Prognostic value of troponins in sepsis: A meta-analysis. Intensive Care Med..

[66] Lim W., Qushmaq I., Devereaux P.J., Heels-Ansdell D., Lauzier F., Ismaila A.S., Crowther M.A., Cook D.J. (2006). Elevated cardiac troponin measurements in critically ill patients. Arch. Intern. Med..

[67] Eggers K.M., Venge P., Lindahl B., Lind L. (2013). Cardiac troponin I levels measured with a high-sensitive assay increase over time and are strong predictors of mortality in an elderly population. J. Am. Coll. Cardiol..

[68] de Lemos J.A., Drazner M.H., Omland T., Ayers C.R., Khera A., Rohatgi A., Hashim I., Berry J.D., Das S.R., Morrow D.A. (2010). Association of troponin T detected with a highly sensitive assay and cardiac structure and mortality risk in the general population. JAMA.

[69] Ahmadian A., Mizzi A., Banasiak M., Downes K., Camporesi E.M., Thompson Sullebarger J., Vasan R., Mangar D., van Loveren H.R., Agazzi S. (2013). Cardiac manifestations of subarachnoid hemorrhage. Heart Lung Vessel..

[70] Mishra R.K., Li Y., De Filippi C., Fischer M.J., Yang W., Keane M., Chen J., He J., Kallem R., Horwitz E.J. (2013). Association of cardiac troponin T with left ventricular structure and function in CKD. Am. J. Kidney Dis..

[71] Dubin R.F., Li Y., He J., Jaar B.G., Kallem R., Lash J.P., Makos G., Rosas S.E., Soliman E.Z., Townsend R.R. (2013). CRIC Study Investigators. Predictors of high sensitivity cardiac troponin T in chronic kidney disease patients: A cross-sectional study in the chronic renal insufficiency cohort (CRIC). BMC Nephrol..

[72] Bansal N., Hyre Anderson A., Yang W., Christenson R.H., deFilippi C.R., Deo R., Dries D.L., Go A.S., He J., Kusek J.W. (2015). High-sensitivity troponin T and N-terminal pro-B-type natriuretic peptide (NT-proBNP) and risk of incident heart failure in patients with CKD: The Chronic Renal Insufficiency Cohort (CRIC) Study. J. Am. Soc. Nephrol..

[73] Scheven L., de Jong P.E., Hillege H.L., Lambers Heerspink H.J., van Pelt L.J., Kootstra J.E., Bakker S.J., Gansevoort R.T., PREVEND study group (2012). High-sensitive troponin T and N-terminal pro-B type natriuretic peptide are associated with cardiovascular events despite the cross-sectional association with albuminuria and glomerular filtration rate. Eur. Heart J..

[74] Keddis M.T., El-Zoghby Z.M., El Ters M., Rodrigo E., Pellikka P.A., Jaffe A.S., Cosio F.G. (2013). Cardiac troponin T before and after kidney transplantation: Determinants and implications for posttransplant survival. Am. J. Transplant..

[75] Baba Y., Kubo T., Nabeta T., Matsue Y., Kitai T., Naruse Y., Taniguchi T., Tanaka H., Okumura T., Yoshioka K. (2025). Prognostic role of high-sensitivity cardiac troponin T in patients with cardiac sarcoidosis: Insights from ILLUMINATE-CS. ESC Heart Fail..

[76] Baba Y., Kubo T., Ochi Y., Hirota T., Yamasaki N., Ohnishi H., Kubota T., Yokoyama A., Kitaoka H. (2023). High-sensitivity Cardiac Troponin T Is a Useful Biomarker for Predicting the Prognosis of Patients with Systemic Sarcoidosis Regardless of Cardiac Involvement. Intern. Med..

[77] Shah F.H., Agrawal S., Tated R.C., Maheta D., Naqvi S. (2025). Novel Biomarkers and Advanced Imaging in Cardiovascular Risk Stratification for Rheumatic Diseases. Cureus.

[78] de Filippi C.R., de Lemos J.A., Christenson R.H., Gottdiener J.S., Kop W.J., Zhan M., Seliger S.L. (2010). Association of serial measures of cardiac troponin T using a sensitive assay with incident heart failure and cardiovascular mortality in older adults. JAMA.

